# Opioid-Induced Glial Activation: Mechanisms of Activation and Implications for Opioid Analgesia, Dependence, and Reward

**DOI:** 10.1100/tsw.2007.230

**Published:** 2007-11-02

**Authors:** Mark R. Hutchinson, Sondra T. Bland, Kirk W. Johnson, Kenner C. Rice, Steven F. Maier, Linda R. Watkins

**Affiliations:** ^1^ Department of Psychology and the Center for Neuroscience, University of Colorado at Boulder, Boulder, CO 80309-0345, USA; ^2^ Discipline of Pharmacology, School of Medical Sciences, University of Adelaide, Adelaide, South Australia, 5005, Australia; ^3^ Avigen Inc., Alameda, CA, USA; ^4^ Chemical Biology Research Branch, National Institute on Drug Abuse, Bethesda, MD, USA

**Keywords:** opioid, glia, cytokine, withdrawal, dependence, analgesia, tolerance, reward, tolllike receptor

## Abstract

This review will introduce the concept of toll-like receptor (TLR)–mediated glial activation as central to all of the following: neuropathic pain, compromised acute opioid analgesia, and unwanted opioid side effects (tolerance, dependence, and reward). Attenuation of glial activation has previously been demonstrated both to alleviate exaggerated pain states induced by experimental pain models and to reduce the development of opioid tolerance. Here we demonstrate that selective acute antagonism of TLR4 results in reversal of neuropathic pain as well as potentiation of opioid analgesia. Attenuating central nervous system glial activation was also found to reduce the development of opioid dependence, and opioid reward at a behavioral (conditioned place preference) and neurochemical (nucleus accumbens microdialysis of morphine-induced elevations in dopamine) level of analysis. Moreover, a novel antagonism of TLR4 by (+)- and (˗)-isomer opioid antagonists has now been characterized, and both antiallodynic and morphine analgesia potentiating activity shown. Opioid agonists were found to also possess TLR4 agonistic activity, predictive of glial activation. Targeting glial activation is a novel and as yet clinically unexploited method for treatment of neuropathic pain. Moreover, these data indicate that attenuation of glial activation, by general or selective TLR antagonistic mechanisms, may also be a clinical method for separating the beneficial (analgesia) and unwanted (tolerance, dependence, and reward) actions of opioids, thereby improving the safety and efficacy of their use.

## INTRODUCTION

The pharmacology and treatment of pain has a very long and tumultuous history. Since the infancy of the use of opium poppy extracts to treat pain around 3500 BC, the search for treatments that provide effective relief from acute and chronic pain has continued to grow at an extraordinary rate. Today, pain still remains a significant public health issue, with two-thirds of patients achieving little to no pain relief from the myriad of currently available pharmacotherapies and dosing regimens[1,2,3,4]. The use of opioid pharmacotherapies produces several rewarding and reinforcing side effects, which result in the drugs' diversion to abuse settings. Unfortunately, a significant side effect in attempting to improve pain patients' quality of life is that some become dependent to the treatments that were prescribed to help them. In recent years, the misuse of opioids has risen drastically[5,6], leaving doctors and patients hesitant to treat pain to the fullest extent[6]. Why is pain still unsuccessfully treated five and a half millennia since opioids were first used as a treatment?

The aim of this review is to highlight that, as researchers and clinicians, we have focused principally on neuronal mechanisms of pain and analgesia, ignoring other potentially powerful modulators of nociception. Three theoretical conclusions have been made that narrowed our understanding of pain processes by limiting how we approached pain research. First, owing to the pain transmission capacity of neurons, these cells have been the primary intentional target of all pharmacotherapies developed to date. Second, it has been concluded that opioids modulate pain solely by acting at neuronal opioid receptors and that opioid antagonists likewise exert their effects solely on neurons. Finally, it is believed that the detrimental (tolerance, hyperalgesia, dependence, and reward) and beneficial (analgesia, cough suppressant) actions of opioids are mediated via very similar and potentially inseparable mechanisms reliant on neuronal opioid receptors.

Here we will demonstrate, by synthesis of prior literature along with new experimental data, that the immunocompetent cells of the central nervous system (glia), their receptors, and their secreted signaling factors are pivotal to a more complete understanding of pain processing and opioid pharmacodynamics. These discoveries have been made by challenging the three historical assumptions mentioned above.

Regarding the first conclusion, glia now have a well-established role in initiating and maintaining increased nociception in response to peripheral nerve injury. More recently, several laboratories have documented that glia can powerfully modulate the analgesic actions of chronically administered opioids[7,8,9,10,11]. Based on such studies, the pharmacological targeting of glia, rather than solely neurons, will be shown to be a novel and as yet clinically unexploited approach for potentially achieving both effective pain control and enhanced efficacy of opioids.

Regarding the second conclusion, we have recently discovered that opioids cause direct glial activation in a nonclassical opioid receptor fashion, via opioid-induced activation of a class of pattern recognition receptors termed toll-like receptors (TLRs)[12]. Intriguingly, in parallel studies, TLRs have been demonstrated to be essential to neuropathic pain states as well[13,14]. Data will be presented here that demonstrate that antagonizing TLRs not only reverses neuropathic pain, but also potentiates morphine analgesia. Finally, regarding the last conclusion, the sum of these discoveries has enabled the molecular separation of the beneficial (classical neuronal opioid receptor-mediated analgesia) and detrimental (glially mediated side effects) actions of opioids.

In the sections to follow, we will first briefly review the role of glia in neuropathic pain. This will be followed by a discussion of more recent evidence indicating that glia are activated in response to opioids and the implications that this opioid-induced glial activation has for analgesia, tolerance, dependence, and reward. Finally, the implications that such glial activation may have for developing novel therapeutic targets will be described, including the novel application of TLR antagonists.

## GLIA AND NEUROPATHIC PAIN

Traditionally, glia (astrocytes and microglia) were viewed as structural supports for neurons and important for maintaining central nervous system (CNS) homeostasis. Glia were long overlooked in pain research due to their lack of axons and their yet-to-be-discovered roles in cell-to-cell communication[15]. While the roles of CNS glia in providing immune surveillance, clearance of debris, and regulation of ionic and chemical composition of the extracellular space have been acknowledged as pivotal to the survival of the host, the actions of glia under varying pain states only came to the attention of pain researchers since the early 1990s[16]. The earliest evidence for a potential role of glia in pain regulation was an associative link between astrocyte activation and neuropathic pain, as drugs that blocked neuropathic pain also decreased glial activation[17].

Upon activation, the functions of microglia and astrocytes change, in that they begin producing and releasing a variety of neuroexcitatory substances, including traditional nociceptive modulators, such as reactive oxygen species, nitric oxide, prostaglandins, excitatory amino acids, and growth factors[18]. A key addition to this list of nociceptive modulators was more recently recognized, namely, proinflammatory cytokines[19,20]. Principal among these are interleukin (IL)-1, IL-6, and tumor necrosis factor-$\alpha$. Spinally, glia are the principal producers of these proinflammatory cytokines, hence, the pivotal importance of glia in explorations of new approaches to clinical pain control[21]. Indeed, spinal glial activation and subsequent release of proinflammatory mediators are now implicated in initiating and maintaining diverse enhanced pain states, including neuropathic pain[19,20].

If glial regulation of neuropathic pain is examined simply as a linear series of events, there are numerous points along this pathway where glia can, at least in theory, be targeted to treat neuropathic pain (Fig. 1). Here we will introduce several experimental treatments that target these steps and discuss their effects on established neuropathic pain. Traditional pain therapies have targeted transmission of the pain signal via neurons (step E; Fig. 1) with limited success[1,2,3,4]. As can be seen in this schematic pathway, merely treating the neuronal component of the pathology will leave the glial component unabated, still attempting to communicate to neurons to propagate pain signals. Perhaps glia are activating neurons via different pathways/intracellular signaling cascades than modulated by drugs directly targeting neurons. Therefore, glial signaling to neurons may act via unique pathways from what has been targeted previously. Perhaps this explanation may elucidate the unfortunate lack of generalized success of current therapies[1,2,3,4].

Perhaps the most tantalizing point in the neuropathic pain pathway, from a clinical point of view, is the initial step wherein glia are triggered to activate (Step A; Fig. 1). Interestingly, a variety of glial activation signals have been identified and which signal(s) initiates glial activation can vary depending on the insult delivered[19,20,21]. As previously noted, several mediators of glial activation have already been well characterized, including neuronally released fractalkine and traditional neuronal nociceptive modulators and transmitters, such as reactive oxygen species, nitric oxide, prostaglandins, excitatory amino acids, substance P, ATP, and growth factors[18], in addition to proinflammatory cytokines[19,20]. In the majority of these cases, known receptor-mediated events have been characterized.

However, a new and exciting initiator and mediator of neuropathic pain are signals relayed by TLRs, specifically TLR2 and TLR4[13,22,23]. TLRs are a family of approximately ten single transmembrane receptors that recognize a diverse range of moieties or “patterns” on exogenous (e.g., lipopolysaccharide [LPS] of gram-negative bacteria such as *E. coli* and Salmonella) and endogenous (e.g., heat shock proteins and cell membrane components released from damaged cells) substances that are considered to be danger signals and, hence, warrant activation of the innate immune system aimed at defending the survival of the host. TLR4 has been extensively characterized, as it is the TLR that recognizes LPS. Binding of agonists to TLRs activates similar downstream intracellular signaling pathways to those previously documented for IL-1 binding to its cognate receptor, resulting in a powerful proinflammatory signal. Indeed, the striking similarity of these pathways is reflected by it being called the TLR/IL-1 signaling cascade[24].

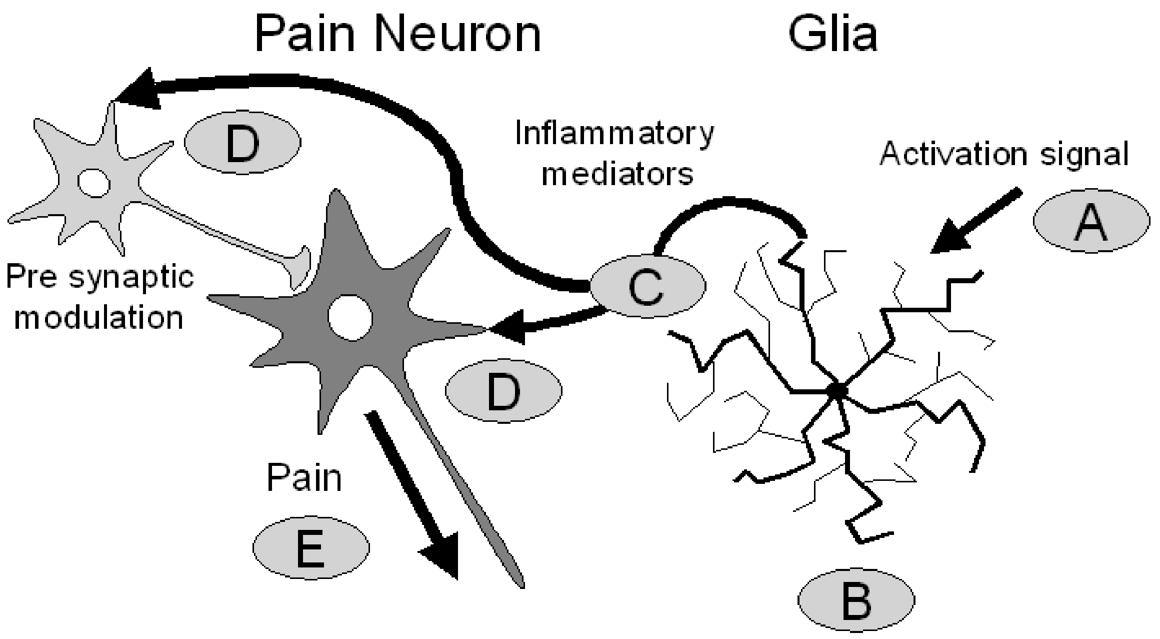

**FIGURE 1.** Schematic of neuropathic pain, highlighting theoretical points (A–E) where pharmacological targets can be designed to treat neuropathic pain to which glia importantly contribute. Step A: An activation or series of activation signals are required to activate glia often mediated via cell surface receptors that can be antagonized. Step B: Glial activation is an all-encompassing term we use for the state in which glia release proinflammatory mediators. This state can be modulated or attenuated, thereby inhibiting various cellular events that block glial activation or its downstream consequences. An anti-inflammatory environment can also be produced, which increases the threshold that an activation signal has to overcome to activate the cells. Step C: Immune inflammatory mediators, such as proinflammatory cytokines, can be neutralized prior to reaching their intended receptor target (pre- and/or postsynaptic) by using soluble receptors (which exist endogenously), neutralizing antibodies, decreasing maturation of cytokines into their active form, or increasing the rate of cytokine degradation. Step D: The action of many glial inflammatory mediators on neurons (pre- and/or postsynaptic) can also be antagonized at neuronal receptor sites. Step E: Included here are the myriad of currently employed neuronal targeted therapies that decrease the neuronal signaling of pain signals (pre- and/or postsynaptic).

As noted above, two TLRs (TLR2 and TLR4) are especially intriguing with regard to pain processing. TLR2 and TLR4 are quite remarkable with regard to the breadth of chemically diverse moieties that activate them. Directly relevant to neuropathic pain is the fact that TLR2 and TLR4 are key TLRs for recognizing and responding to endogenous danger signals that are released by damaged, dying, and dead neurons and other cells (host DNA and RNA, heat shock proteins, cell membrane components, etc.) and more general aspects of tissue injury (plasma proteins, extracellular matrix degradation products, etc.)[22,23]. TLR knockout and knockdown studies have demonstrated that both TLR2 and TLR4 are involved in animal models of neuropathic pain, as knockouts and/or knockdowns of each suppress the development and/or maintenance of nerve injury–induced allodynia (pain resulting from a normally nonpainful stimulus)[22,23]. We have extended these findings from transgenic animals and subacute knockdowns to normal adult rats by demonstrating that acute intrathecal administration of a selective TLR4 antagonist suppresses well-established neuropathic pain induced by chronic constriction injury (Fig. 2).

Taken together with the knockout/knockdown studies, these data demonstrate ongoing stimulation of TLRs, likely by injury-induced endogenous danger signals. As (a) peripheral nerve injury leads to protracted expression of heat shock proteins in proximal axons of damaged sensory neurons[26] and degradation of presynaptic terminals, and (b) nerve degeneration in the CNS occurs remarkably slowly, taking months to years[27], it is clear that endogenous danger signals created as a result of nerve injury could produce perseverative activation of at least TLR2 and TLR4 and, thereby, a perseverative drive for maintaining neuropathic pain. Thus, targeting step A (Fig. 1) of glial activation may have potential clinical utility.

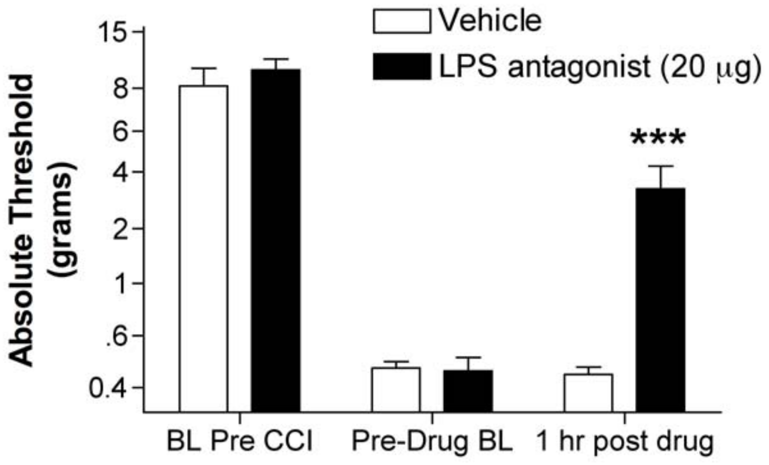

**FIGURE 2.** Chronic constriction injury (CCI)–induced allodynia is reversed by acute blockade of TLR4**.** Intrathecal delivery of 20 μg of LPS antagonist (LPS from *msbB* mutant *E. coli*; Invivogen, San Diego, CA) acutely reverses allodynia induced by CCI of the sciatic nerve (n = 4 per group). This LPS antagonist is a mutant form of LPS that lacks the myristoyl fatty acid moiety of the lipid A and displays 1,000- to 10,000-fold reduction in activation of NF-κB and acts as an antagonist of TLR4[25]. BL (baseline) pre-CCI Von Frey testing of thresholds occurred the day of CCI surgery of the left sciatic nerve. Allodynia developed across the following 10 days with maximal allodynia achieved and maintained from this point for a further 21 days. Predrug BL thresholds were assessed the morning of the test day. Acute intrathecal delivery via the lumbar approach of vehicle and drug was conducted under brief isoflurane anesthesia. Von Frey testing was conducted 1 h postdrug. All testing was conducted blinded to treatment group by two separate experimenters[12].

While TLRs provide a novel and intriguing target for neuropathic pain control, new TLR antagonists would need to be developed before such a strategy could have clinical utility. For example, the selective TLR4 antagonist used in the study depicted in Fig. 2 is not likely to be a viable clinical candidate due to its lack of penetrance of the blood brain barrier if given systemically. In addition, given that both TLR2 and TLR4 signal the presence of endogenous danger signals, it would be predicted that antagonizing either would have beneficial effects and that the ideal antagonist may be one that blocks both.

Given these considerations, we sought a blood brain barrier permeable, small molecule class that could block TLR4 and/or TLR2. Our attention was drawn to the work of Hong et al.[28,29,30] and Lu et al.[31] that demonstrated blockade of LPS activity by the opioid receptor antagonist naloxone. Importantly, relating to our desired application, naloxone was documented to block microglial activation by LPS[29].

Intriguingly, Liu et al., from Hong’s group[29], found that the actions of naloxone on LPS-induced microglial activation were not mediated by classical neuronal opioid receptors as both the opioid active (–)-isomer and the opioid inactive (+)-isomer of naloxone-exerted identical inhibitory effects on LPS microglial activation. The observation that (+)-naloxone was equally effective was a critical observation as it means that naloxone must be acting through some receptor other than the classical opioid receptor, which only bind (–)-isomers. One possibility was that naloxone bound TLRs in addition to classical opioid receptors. To test this, we sought to determine if (+)-naloxone could block LPS-induced activation of TLR4. Using a TLR4 stably transfected cell line (Invivogen) with a stable cotransfection of an NF-κB reporter gene (secreted embryonic alkaline phosphatase; SEAP), we found a significant noncompetitive antagonism of LPS activity at TLR4 by (+)-naloxone (Fig. 3). The antagonism was concluded to be noncompetitive owing to the reduction in $E_{max}$.

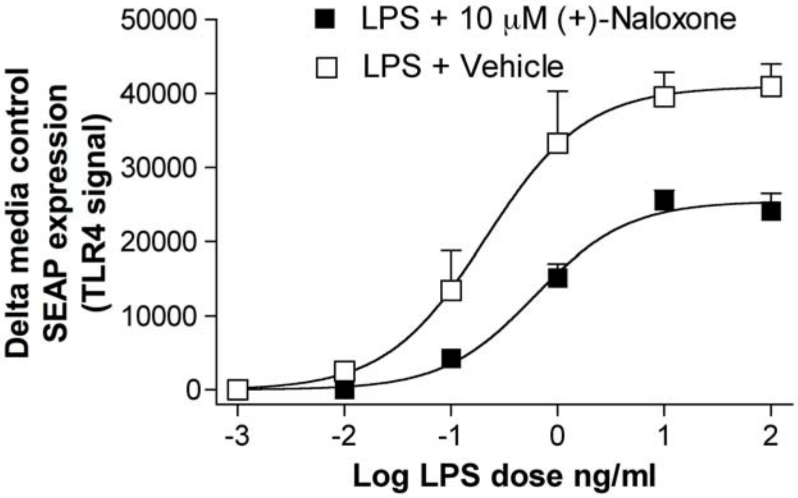

**FIGURE 3.** (+)-Naloxone noncompetitively antagonizes activation of TLR4 by LPS. A TLR4 (cloned human receptor) stably transfected cell line was incubated for 24 h with LPS, with or without 10 μ*M* (+)-naloxone. SEAP expression was quantified as an indirect measure of TLR4 activation. LPS alone produced an $EC_{50}$ of 0.21 ng/ml with an $E_{max}$ of 40,956 (arbitrary fluorescence units, change from media and (+)-naloxone alone controls). (+)-Naloxone (10 μ*M*) produced a significant threefold increase in the EC50 and a 40% reduction in the $E_{max}$, while having no effect on basal unstimulated SEAP expression and, therefore, no effect on basal NF-κB activation[12].

The success of (+)-naloxone in antagonizing TLR4 receptors *in vitro* led us to test whether it could acutely reverse CCI-induced allodynia, similar to the reversal observed following intrathecal administration of a known TLR4 antagonist (mutant LPS antagonist) (Fig. 3). Indeed, (+)-naloxone also reliably reversed CCI-induced allodynia 90 min following a systemic administration (60 mg/kg s.c.; Fig. 4). These (+)-naloxone data demonstrate two vital points. First, blood brain barrier permeable small molecules can be used to antagonize TLR4 activity *in vivo*. Second, TLR4 antagonism by this small molecule successfully reverses CCI-induced allodynia (step A; Fig. 1), demonstrating a novel receptor antagonistic action of (+)-naloxone and re-emphasizing the pivotal role of TLR4 receptors in neuropathic pain. A fortunate characteristic of (+)-naloxone related to its potential clinical utility is that if breakthrough opioid pain medication was required in a routine or emergency situation, opioid analgesia would be unaffected owing to the lack of opioid activity of the (+)-isomer. Importantly, such effects are not restricted to (+)-naloxone, as we have demonstrated similar properties of another (+)-isomer of an opioid antagonist[32]. (+)-Naltrexone delivery intrathecally or systemically reversed established CCI-induced allodynia, as did (–)-naltrexone. From this it would appear that naltrexone can reverse neuropathic pain by nonstereoselectively antagonizing TLR4 receptors, acting in a nonclassical opioid fashion as naloxone had been shown previously to do. The potential clinical utility of (–)-naltrexone or (–)-naloxone in this indication as sole treatment of neuropathic pain would be unwise as breakthrough pain treatment with opioids would be rendered ineffective. The (–)-isomer antagonist obviously will not have an indication for cotherapy with chronic opioids.

The application of putative TLR4 antagonists to treat neuropathic pain emphasizes the exciting clinical opportunity potentially provided by this new approach. However, as mentioned previously, the mode of glial activation that results in enhanced pain can vary depending on the insult delivered[19,20,21] and, therefore, it is possible that some neuropathies may develop independent from TLR4 agonism. Thus, when the initiating signal for glial activation is targeted, this suggests that effective treatment will depend on which glial activating signal(s) are responsible for the pain pathway. A broader therapeutic approach would be to inhibit or attenuate existing glial activation and/or products released by activated glia (step D; Fig. 1). Examples of such drugs that have efficacy in reducing animal models of

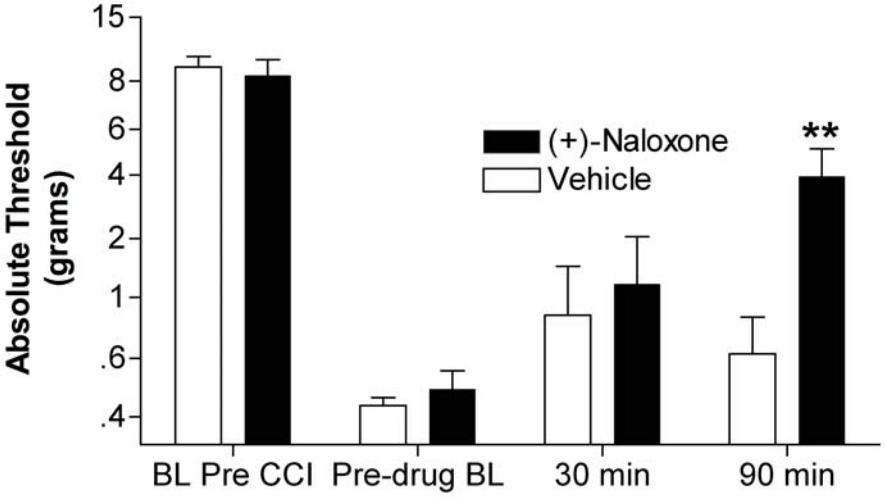

**FIGURE 4.** Systemic (+)-naloxone acutely reverses CCI-induced allodynia. (+)-Naloxone (60 mg/kg s.c., kindly supplied by NIDA drug repository) reversed established allodynia 90 min, but not 30 min, following drug administration (n = 5 per group). BL pre-CCI Von Frey testing of thresholds occurred the day of CCI surgery of the left sciatic nerve. Allodynia developed across the following 10 days with maximal allodynia achieved and maintained from this point for a further 21 days. Predrug BL thresholds were assessed the morning of the test day. All Von Frey testing was conducted blinded to treatment group by two separate experimenters 30 and 90 min postdrug admin[12].

neuropathic pain include propentofylline[33], pentoxifylline[34], AV411 (ibudilast)[35], and AV333 (IL-10 gene therapy)[36,37]. For each, neuropathic pain is reversed, as it was for (+)-naloxone. Also like (+)-naloxone, all of these treatments return the animal toward normal basal pain responsivity, rather than producing analgesia. Therefore, all of these treatments are specifically antiallodynic and/or antihyperalgesic, leaving basal nociception unaffected.

As discussed previously, the inflammatory and pronociceptive mediators released by glia in their activated state are numerous. Therefore, clinically antagonizing or neutralizing each mediator (targeting step C and/or D) has its limitations, as inhibiting the actions of each of these numerous mediators individually may be too great a task. However, proinflammatory cytokines appear to be a key linchpin in glial enhancement of pain, as neutralizing the action of principal proinflammatory cytokines (IL-1, IL-6, tumor necrosis factor-α; step C, Fig. 1) or antagonizing their receptors (step D; Fig. 1) has proven a successful strategy for preventing and reversing neuropathic pain[38,39].

As with all pharmacotherapies, there is a cost-benefit ratio resulting from likely side effects of treatment. When examining the stages of neuropathic pain to pharmacologically target (Fig. 1), step A is the most attractive. In the case of neuropathy resulting from TLR agonism, treatment of this pathology with selective TLR antagonists will only reduce the ability of the innate immune system to respond to new infection or challenges that are solely recognized by the specific TLR(s) that is/are pharmacologically blocked. As there are redundant pathways utilized by the immune system to recognize and respond to pathogens, blockade of select TLR(s) would not be expected to pose significant risks. This can be demonstrated in the clinic in the case of the well-tolerated chronic use of (–)-naltrexone, which our data suggests will antagonize TLRs.

Targeting glial activation events after step A (B–D) has the potential to have broader nonselective actions on either peripheral immune responses (likely with targeting steps C and D) and normal glial functions. These changes may compromise host immune defense or maintenance of CNS homeostasis. While these are theoretical issues, AV411 (ibudilast) has been used for over 20 years in Asia, for a different indication, and has an excellent safety record[35]. Therefore, while there are important issues in developing and employing these pharmacotherapies, they are not insurmountable. It is our belief that the benefits of this course of treatment will likely outweigh these potential costs.

As a whole, all these studies highlight the multiple novel targets for new pharmacotherapies when glia are considered as key mediators of neuropathic pain. We have confirmed the role of TLR4 in mediating neuropathic pain and our data have provided the exciting opportunity for the development of (+)-opioid antagonists to treat neuropathic pain. This development could provide a novel drug class to help treat the untreatable pain.

## OPIOIDS SIMULTANEOUSLY INDUCE ANALGESIA AND GLIAL ACTIVATION

A parallel line of research in our laboratory focuses on opioid-induced glial activation. We[21], among others[10], have drawn parallels between the glial activation observed in response to peripheral neuropathy and that observed following chronic opioid exposure. The understanding of glial involvement in neuropathic pain and opioid pharmacodynamics has grown together, such that they are now indelibly intertwined. Our discovery that opioid agonists activate TLR4 and opioid antagonists ***nonstereoselectively*** block TLR4 activation developed in parallel to our investigations into the mechanisms underlying opioid-induced glial activation. It appears that TLRs may lie at the intersection between neuropathic pain and opioid-induced glial activation. Thus, as will be reviewed below, the exciting potential exists that (+)-opioid antagonists may be uniquely positioned to suppress simultaneously neuropathic pain, suppress opioid-induced glial activation, and yet not compromise the pain-suppressive effects of opioids agonists on neurons.

Since the discovery of morphine modulation of T-cell function by Wybran et al. in 1979[40], an extraordinary amount of work has been focused on characterizing the influence that opioid exposure has on the functioning of the immune system in its traditional role of host defense. However, the impact that the activation status of immunocompetent cells has on opioid actions has only been recently recognized. While modulation of peripheral immune cells function by opioids is very important to understanding host defense, these cells are not as likely as glia to have a profound effect on opioid pharmacodynamics. The immunocompetent cells that are perfectly anatomically located to mediate effects on opioid analgesia are the glia of the dorsal root ganglia, spinal cord, and brain.

The notion that glia can modulate opioid actions is not a new one, as it was first postulated in the late 1980s, long before glia were even recognized as contributing to neuropathic pain[41,42,43]. However, it took the groundbreaking research of Song and Zhao[11] to both associatively and then causally link opioid-induced glial activation with the development of tolerance. Song and Zhao demonstrated that (a) chronic morphine administration causes glial activation in spinal cord and brain, and (b) coadministration of morphine with a glial activation inhibitor resulted in a maintenance of analgesic efficacy and a corresponding reduction of glial activation. Theirs were the first lines of evidence that morphine analgesia could be modified by opioid-induced glial activation. Interestingly, glial activation induced by other phenomena, such as neuropathic pain[9,34] or LPS administration[44,45], also reduces the analgesic efficacy of morphine, producing a state akin to “naïve” tolerance. Such studies led to the recognition that at least several of the same products of activated glia that produce neuropathic pain also oppose morphine analgesia[45]. Since 2001, several other research groups have confirmed and refined this hypothesis, demonstrating that, following chronic administration, morphine tolerance and morphine-induced hyperalgesia are produced, at least in part, as a consequence of glial activation[8]. One mechanism that has been proposed to account for such effects is via nitric oxide–induced p38 MAPK activation[46,47], with downstream up-regulation of proinflammatory cytokines[7,10]. IL-1, in turn, opposes morphine analgesia[48].

If these discoveries are taken in the context of the schematic diagram used previously to illustrate neuropathic pain (Fig. 1), it would appear that morphine was acting not only at classical opioid receptors on nociceptive neurons (step E, Fig. 1), but also as a glial activation signal (step A, Fig. 1) producing the same, or at least a similar, cascade of events that results in increased nociception. The sum of morphine's neuronal antinociceptive activity and its pronociceptive glial activation results in a net reduction in analgesia[49]. Moreover, since glial activation increases with chronicity of opioid treatment, this process produces a gradually increasing opposing process, significantly contributing to the behavioral phenomenon of analgesic tolerance. It also seems likely that opioid-induced glial activation contributes significantly to the atypical allodynia and hyperalgesia that results from chronic opioid administration[49]. We have also addressed the question of how rapidly opioid-induced glial activation occurs following morphine administration and have found that IL-1, as well as other proinflammatory cytokines, opposes morphine analgesia within minutes after either systemic or intrathecal administration[49].

The parallels between neuropathy- and opioid-induced glial activations can also be explored by using agents that reverse nerve injury–induced allodynia so as to define whether these same agents modulate morphine analgesia as well. We and others have demonstrated that agents that oppose neuropathic pain, either by suppressing glial activation or by neutralizing or antagonizing proinflammatory glial products, also oppose glial attenuation of both acute and chronic morphine analgesia[49]. The efficacy of morphine can be potentiated by targeting opioid-induced glial activation (step B, Fig. 1), or by neutralizing (step C, Fig. 1) or antagonizing (step D, Fig. 1) the action proinflammatory cytokines[49]. Again, these data demonstrate the significant parallels between glial activation that occurs as a result of neuropathic pain and opioid exposure.

All studies published to date have investigated glial activation by morphine, the prototypical opioid. However, for modulation of opioid analgesic efficacy by glial activation to be globally relevant, this phenomenon should be generalizable to other clinically relevant opioids. This is, in fact, the case as glial activation in response to another 4,5-epoxymorphinan, oxycodone[50], and the structurally disparate synthetic 3,3-diphenylpropylamine opioid, methadone[49], each compromise the resultant analgesia.

Interestingly, etorphine does not appear to produce glial activation[51]. This is consistent with the possibility that the activation of glia is not mediated via a classical "neuronal-like" opioid receptor. We have documented the involvement of a nonclassical opioid receptor in glial activation using (+)-methadone. (+)-Methadone, which possesses no classical opioid receptor activity, causes significant glial activation, allodynia, and hyperalgesia, as well as up-regulation of proinflammatory cytokine mRNA, protein and release[21]. Other investigators have also demonstrated nonopioid morphine-induced hyperalgesia in triple opioid receptor knockout animals[52] and immunomodulation generally[53,54]. Glia do express classical opioid receptors[55]. However, given the compelling data reviewed here, it seems likely that the immunomodulation resulting from opioid exposure is not mediated by these receptors. Previous findings of opioid effects on glia will need to be reassessed with nonclassical opioid receptors in mind.

This brings us back to step A of glial activation as discussed previously (Fig. 1). Once again, prior literature was instructive regarding potential mechanisms and targets. Wu et al.[44,56,57] have demonstrated that (+)-naloxone possesses a glial-dependent protective effect over a phenomenon called "morphine-induced antianalgesia". In morphine-induced antianalgesia, prior administration of low-dose morphine attenuates subsequent analgesia induced by a later higher dose of morphine. The prior low morphine dose produces a state akin to "naïve" tolerance produced by a glially dependent mechanism, likely via production of proinflammatory cytokines. Owing to the prevention of antianalgesia by (+)-naloxone[57] and to our experience of (+)-naloxone's antiallodynic capacity, we examined the possibility that (+)-naloxone could potentiate morphine analgesia by blocking morphine-induced glial activation and consequent increase in antianalgesic proinflammatory cytokines. Indeed, (+)-naloxone significantly potentiated both acute and chronic morphine analgesia[58].

Taken together with our previous TLR4 cell line data demonstrating that (+)-naloxone blocked TLR4 activation, the above findings suggest that opioids such as morphine may be as-yet-unrecognized agonists of TLR4. If this were true, it would provide a novel mechanism by which opioids could activate glia. To test this idea, we screened several clinically employed opioid agonists on the same TLR4 cell line described previously, and all were found to be TLR4 agonists. These opioid TLR4 agonists included (+)- and (–)-morphine, (+)- and (–)-methadone, oxycodone, buprenorphine, fentanyl, and pethadine/meperidine, among others.

### Like Neuropathic Pain, Clinically Employed Opioids Activate Glia via TLRs

By targeting opioid-induced activation of glial TLRs, we are able to block this detrimental aspect of glial activation from even progressing past step A (Fig. 1) that contributes to opioid-induced tolerance, allodynia, and hyperalgesia. In doing so, we believe the beneficial neuronally induced opioid analgesia is unhindered by opioid-induced glial activation. Early evidence indicates that acute opioid exposure increases the expression of at least TLR4 at both the transcriptional and translational levels (Hutchinson et al., unpublished observations). Ongoing studies are investigating the changes in expression of TLRs following chronic opioid administration.

The significance of the discovery of opioid-TLR4 activity should not be underestimated, as it would appear that at least TLR4 is responsible for initiating a component of opioid-induced glial activation that contributes significantly to the unwanted pronociceptive side effects of opioid administration. Moreover, TLRs provide a molecular point at which the paths of neuropathic pain and pronociceptive actions of morphine cross. Therefore, lessons learned from neuropathic pain and atypical nociceptive changes resulting from morphine exposure may be mutually beneficial to each research field.

## OPIOID-INDUCED GLIAL ACTIVATION AND OPIOID ABUSE LIABILITY

The importance of defining how broadly glia impact opioid actions led us to investigate the role that opioid-induced glial activation may have in nonanalgesic activity of opioids, such as dependence and reward. Immune involvement, as a whole, in morphine dependence was first demonstrated by Dafny et al.[59], who found that several nonselective immunosuppressive treatments ameliorated morphine withdrawal behaviors. These studies were conducted prior to an appreciation of the importance of glial cells. A significant concern surrounding Dafny's research was the severity of the immunosuppressive treatments used and the possibility that the animals were too sick to even display withdrawal behaviors[60,61]. Such concerns led to the end of this line of research for over 10 years. However, one intriguing aspect to Dafny's research was that his use of systemically administered immunosuppressants would have also suppressed glial activation. If true, this would provide yet another novel therapeutic indication for attenuation of glial activation. A second thread of evidence was the work of Johnston et al.[7], which suggested glial involvement in morphine withdrawal induced pain enhancement, as this enhanced nociception could be blocked by IL1ra or IL-10. As the work of Johnston et al. utilized an intrathecal route of morphine administration[7], these spinally mediated events begged the question of whether dependence/withdrawal at supraspinal sites may involve glia as well.

To readdress whether immunocompetent cells, specifically glia, may be involved in morphine withdrawal, we explored whether morphine dependence/withdrawal might be suppressed by AV411 (ibudilast), a blood brain barrier permeable glial activation inhibitor that suppresses neuropathic pain[35]. We found that coadministration of AV411 with an escalating dependence regimen of morphine significantly reduced naloxone-precipitated withdrawal behaviors[35]. Moreover, there was a corresponding reduction in glial activation in brain nuclei associated with opioid action[50]. In a separate experiment, AV411 was found to protect against previously established dependence and spontaneous withdrawal, as reflected by suppression of withdrawal-induced spontaneous activity levels and weight loss[50]. These data implicate, for the first time, opioid-induced glial activation in the development of morphine dependence and precipitation of withdrawal behaviors. If it is indeed true that glia, and specifically morphine-induced glial activation via TLR4, importantly contribute to opioid dependence/withdrawal, then (+)-naloxone would also be expected to attenuate (–)-naloxone–precipitated withdrawal in morphine-dependent animals. Indeed, this is true[58], as coadministration of (+)-naloxone significantly reduced withdrawal behaviors and attenuated morphine-induced weight loss. Therefore, TLR4 is also implicated in the development of morphine dependence and is a point at which the mechanism of the detrimental side effects of opioids initiates.

Given the success of separating the beneficial and detrimental actions of opioids by targeting glial activation, the next extension of the research was to investigate the rewarding and reinforcing actions of morphine. Work by Narita et al.[62] implicated glial activation in the rewarding properties of both morphine and methamphetamine in mice, as measured by conditioned place preference. They established that the glial activation inhibitor, propentofylline, reduced conditioned place preference induced by morphine and methamphetamine[62]. Down-regulation of glial glutamate transporters were implicated as playing an important role in their model of glial involvement in morphine reward[63,64]. We wished to address the issue of glial involvement in morphine reward from both a neurochemical and behavioral standpoint. As such, we assessed both morphine-induced elevations in dopamine levels in the nucleus accumbens, a neurochemical hallmark of reward for opioids and other drugs of dependence; and morphine-induced conditioned place preference, a behavioral hallmark of reward. In both cases, coadministration of AV411 and, hence, attenuation of opioid-induced glial activation, resulted in a significant reduction in morphine reward[35,50]. An extension of this hypothesis is that TLRs mediate the detrimental reinforcing and addictive actions of morphine. However, in both dependence and reward traditional neuronal opioid signals are, not surprisingly, still required for these responses to be achieved as a pure TLR4 signal is not rewarding alone[65]. It remains to be assessed if (+)-naloxone possesses the same antirewarding action when coadministered with morphine. Given Narita et al.[62] demonstrated glial activation following methamphetamine, which is structurally disparate to opioids, the potential common recruitment of pattern recognition receptors such as TLRs is a tantalizing and as yet untested hypothesis. If true, then TLR agonism and subsequent glial activation may be a common mechanism of abuse liability for several structurally diverse classes of compounds.

## CONCLUSIONS

By examining the role of glia in neuropathic pain and opioid actions, three important clinically relevant discoveries have been made. First, TLR-dependent glial activation is pivotal to the maintenance of neuropathic pain. Second, TLR-dependent opioid-induced glial activation is fundamental to reducing morphine analgesia, producing dependence, and producing morphine reward. Finally, antagonism of TLRs or attenuation of glial activation in both neuropathic pain and during opioid exposure reverses allodynia and reduces unwanted opioid side effects, while maintaining opioid analgesic efficacy, respectively. Importantly, the negative side effects of opioids can be separated from the beneficial actions by targeting opioid-induced glial activation using blood brain barrier permeable pharmacotherapies such as AV411 or (+)-opioid antagonists[35]. There is immense clinical utility for such nonopioid treatments for chronic pain and opioid dependence, as they would be predicted to have no abuse liability.

The evidence that methamphetamine induces glial activation also highlights the possibility that glial activation may be a common link in the rewarding capacity of several abused compounds. Therefore, ongoing glial activation may potentially provide a predictor for a patient’s drug abuse liability. Examples of patient populations where this may pertain include HIV/AIDS, stress, and depression, just to name a few. In all these cases, drug abuse is of considerable concern. Including glia in future pain and drug dependence hypotheses will provide a plethora of novel treatment opportunities and hopefully increase the success rate and safety of current pharmacotherapies.

## ACKNOWLEDGMENTS

Avigen Inc, International Association for the Study of Pain International Collaborative grant, American Australian Association Merck Company Foundation Fellowship, National Health and Medical Research Council of Australia CJ Martin Fellowship (ID 465423), and NIH Grants DA015642, DA017670, and DE017782.
